# 2311. Predictive cut-off values for anti-N and anti-S antibodies for COVID-19 risk identification

**DOI:** 10.1093/ofid/ofad500.1933

**Published:** 2023-11-27

**Authors:** David E Rebellón Sánchez, Julio Llanos-Torres, Eric Tafurt, Fernando Rosso

**Affiliations:** Fundacion Valle de Lili, Cali, Valle del Cauca, Colombia; Fundación Valle del Lili, Cali, Valle del Cauca, Colombia; Fundación Valle del Lili, Cali, Valle del Cauca, Colombia; Fundación Valle del Lili, Cali, Valle del Cauca, Colombia

## Abstract

**Background:**

The role of anti-N and anti-S antibody tests as a monitoring strategy for identifying patients at risk of developing COVID-19 is not clearly elucidated. Our study explored in a cohort of hospital workers whether the positivity of these antibodies linked to infection and/or vaccination was associated with a decrease in the risk of infections and sought to estimate a predictive cut-off point for the development of COVID-19 in the first 6 months of measurement.

**Methods:**

A prospective observational study was conducted among hospital workers at a university hospital in Cali, Colombia. Measurements of COVID-19 antibodies were taken before the onset of the third, fourth, and fifth waves of COVID-19 cases. Anti-N and Anti-S total antibodies were measured using Elecsys® Anti-SARS-CoV-2 Immunoassay (Roche).

**Results:**

We include 480 participants, 71.8% females and median age of 32 years (IQR: 27-39); 60.62% were healthcare workers involved in the care of COVID-19 patients. It was found that having a history of COVID-19 (aRR 0.49 (95% CI 0.34-0.70)), being vaccinated in the last 6 months (aRR 0.13 (95% CI 0.07-0.24)), having positive anti-N antibodies in the last 3 months (aRR 0.62 (95% CI 0.44-0.87)), and having positive anti-S antibodies in the last 3 months (aRR 0.55 (95% CI 0.31-0.97)) were associated with a lower risk of developing COVID-19. A cutoff point ≤ 150 COI for anti-N levels and ≤1,900 BAU/mL for anti-S levels was found to have a better predictive performance. The chosen cutoff point for anti-N achieved a sensitivity of 98.2%, with a specificity of 12.4%, a negative predictive value (NPV) of 98.5%, and a positive predictive value (PPV) of 10.2%, with an AUC of 0.55 for predicting COVID-19 in the next 6 months. Having anti-S antibodies ≤ 1900 BAU/mL was found to have a sensitivity of 58.1%, specificity of 55.5%, NPV of 85.3%, PPV of 22.9%, and an AUC of 0.63.

**Conclusion:**

Our study suggests that having a positive anti-N or anti-S antibodies result may be associated with a lower risk of developing COVID-19. We also identified predictive cut-off points for anti-N and anti-S antibody levels that may be useful in identifying individuals at a higher risk of developing COVID-19 in the next 6 months. However, further studies are needed to confirm these findings and determine their generalizability to other populations.

**Disclosures:**

**All Authors**: No reported disclosures

